# Correlation of *Lawsonia intracellularis* positivity in quantitative PCR and herd factors in European pig herds

**DOI:** 10.1186/s40813-021-00192-4

**Published:** 2021-01-22

**Authors:** Mirjam Arnold, Annelies Crienen, Hanny Swam, Stephan v. Berg, Rika Jolie, Heiko Nathues

**Affiliations:** 1 Clinic for Swine, Department for Clinical Veterinary Medicine, Vetsuisse Faculty, University of Bern, Bern, Switzerland; 2 Center for Diagnostic Solutions, MSD AH Boxmeer, Boxmeer, The Netherlands; 3 MSD Animal Health, Munich, Germany; 4 Merck Animal Health, Madison, NJ 07940 USA; 5 Department for Clinical Veterinary Medicine, Farm Animal Clinic, Vetsuisse-Faculty, University of Bern, Bremgartenstrasse 109 a, CH-3012 Bern, Switzerland

**Keywords:** Control, Europe, Porcine proliferative enteropathy, Risk factor, Swine

## Abstract

**Background:**

*Lawsonia intracellularis* is causing diarrhea, poor growth and sudden death in pigs. It can be found in most pig populations leading to large economic losses worldwide. Many potential risk factors for the occurrence of disease or seropositivity have been described. The current study therefore focused on herd characteristics in European countries associated with direct detection of the pathogen determined by quantitative polymerase chain reaction.

**Results:**

A median number of less than 30 nursery pigs per pen was correlated to less positive nursery pigs (*p* < 0.01) and generally less samples positive per herd (*p* < 0.05) as well as a lower median of genome equivalents determined per herd (*p* < 0.05). Routine use of zinc oxide at/ around weaning, which was mentioned by 41.0% of all farmers, was correlated to higher number of positive nursery pigs (*p* < 0.01) as well as higher median genome equivalents determined per herd (*p* < 0.05). Slatted flooring of more than 78.0% of the surface in nursery units was correlated to lower number of positive animals (*p* < 0.05) and a lower median of genome equivalents per herd (*p* < 0.05). A weight of more than 7.8 kg at weaning was correlated to a higher number of positive growing pigs (*p* < 0.05) as well as general higher number of positive samples/ herd (*p* < 0.01).

**Conclusions:**

Weaning and subsequent accommodation of nursery pigs seem to be of particular importance in prevention of infection with *Lawsonia intracellularis* and the spread of the pathogen within the herd.

**Supplementary Information:**

The online version contains supplementary material available at 10.1186/s40813-021-00192-4.

## Background

*Lawsonia (L.) intracellularis* is the causative agent of porcine proliferative enteropathy (PPE). Clinical signs and affected age categories vary depending on whether the acute or chronic from precedes [1]. The clinical appearance consists mainly of diarrhea, poor growth and increased mortality, whereby the widespread of the pathogen causes a large impact on pig production worldwide [2]. Herds which are completely negative in both, serology and direct pathogen detection like quantitative polymerase chain reaction are rare [3]. Antibodies against the ubiquitous pathogen are found particularly in fattening or breeding animals, indicating prior contact to the pathogen [3, 4]. However, contact to the pathogen must not necessarily always led to clinical disease [5]. Shedding of the pathogen is most often described in growing pigs [3, 6], whereby its concentration correlates with the severity of lesions in histopathology or immunohistochemistry [7, 8]. Multiple risk factors for disease or occurrence of the pathogen are discussed. British and Irish herds for example are routinely considered as seropositive if all age categories are located at the same site (single-site farms) [9]. However, if these age categories are separated by two- or multiple-site systems, antibodies in grower pigs are reported to be less likely [10, 11]. On the other hand lower prevalence were reported in herds respecting the rules of ‘all-in all-out’ [12] or routinely destocking the entire building. Destocking of selected pens on the other hand once showed such association [13] and then again not [14]. Although a general- or batch-wise ‘all-in all-out-‘ system may suggest better hygiene, few studies have demonstrated a significant effect of cleaning and/or disinfection itself on the serological status or clinical appearance of PPE [15, 16]. Antibiotics and zinc amino acid complexes are reported to reduce the number of animals with, and severity of lesions as well as faecal shedding [17, 18]. However, also correlation of reduced disease prevalence and the absence of post-weaning medication is described [14]. The extent to which the type of flooring influences the occurrence of PPE and seropositivity is a matter of controversy. For instance fully slatted, or meshed floor in nursery is considered a risk factor for an PPE outbreak [14]. The suspected cause is an accumulation of faeces in the pen and difficulties in cleaning slatted floors [14, 16]. Another study could only detect increased seropositivity in herds raising more than 65% of pigs on concrete slats [19]. The presence of straw in addition had either no effect, or was even correlated to the absence of the disease [13, 14]. Beside hygiene and biosecurity, other risk factors such as serological status of the breeding pigs, herd size, or number of pigs entering the farm are discussed but not consistently proven [19].

Many potential risk factors for the onset of PPE or the presence of antibodies against *L. intracellularis* have been described. However, which factors are associated with an increased occurrence of the pathogen, i.e. which favour a spread inside the herd, are still rarely described. Furthermore, possible differences in husbandry and management systems between countries make it difficult to compare the detected risk factors and their importance. The aim of this study was therefore to identify herd characteristics across countries, correlating with the direct detection of *L. intracellularis* in pig herds.

## Methods

### Study design

In a previously published study dealing with the prevalence of *L. intracellularis* in Europe, faecal samples from 24 herds per participating country were obtained [3]. Countries included were Germany (DE), Denmark (DK), Spain (ES), France (FR), Netherlands (NL), and the United Kingdom (UK). In each herd, a questionnaire, which can be viewed in full as Additional file 3, focussing on herd structure, feeding, hygiene, immunoprophylaxis, treatments and the last occurrence of diarrhea was filled in by the veterinarian, who later carried out the sampling. Samples were taken from 15 nursery- (NP: 10–25 kg), 15 growing- (GP: 25–40 kg) and 15 finishing pigs (FP: 40–100 kg) per herd. Samples were sent to one laboratory and were analysed by quantitative real time polymerase chain reaction (qPCR). Further details on in- and exclusion criteria, collection and analysis of the samples and herds can be found elsewhere [3].

### Statistical analysis

After collection, laboratory results were summarized on herd level applying five outcome variables: ‘Number of positive samples per herd’, ‘Median genome equivalents (GE)/μl per herd’ and ‘Number of positive NP, GP or FP /herd’. Subsequently data were transferred to a statistic software (NCSS 12 Statistical Software (2018) USA,) and descriptive statistics were performed. Some answers, especially metric ones were then grouped into factor levels due to their high diversity (detailed description see Additional file 1). The basis therefore was the distribution in descriptive statistics and biological plausibility. In case requested parameters applied to nearly all or no farm, the variables were excluded from further statistical analysis, if no meaningful grouping was possible. Furthermore, a very high variability of answers concerning antimicrobial use within the last 6 months before sampling and vaccination programs, excluding vaccination against *L. intracellularis,* meaningful grouping and thus a further statistical analysis of these data were not possible. For each of the five outcome variables (Positive samples/herd, Median GE/μl, Positive NP/herd, Positive GP/herd, Positive FP/herd), a univariable hypothesis test against each of the independent variables was performed. Categorical variables were tested with either Wilcoxon Rank Sum tests (two factor levels) or Kruskal Wallis ANOVA- including Dunn’s tests (three or more factor levels), to assess differences in the disease outcome among the corresponding factor levels. All variables with *p* < 0.05 in these univariable test were included in a correlation analysis (Spearman Correlations). If the absolute correlation coefficient was higher than 0.6, the variable with the lower *p*-value in the univariable test was included in a multivariable model. As the number of significant variables remained high after screening the variables for collinearity, three subgroups ‘Environment’, ‘Internal Biosecurity’ and ‘Animal related’, were created based on biological plausibility and distribution of variables. Subsequently, multivariate analysis to account for the effect of each selected variable on the disease outcome simultaneously, was performed. For each subgroup, a generalized linear mixed model was used for multivariable analysis, each with country as random effect. A manual forward selection starting with the smallest p- value was applied with usage of the three sub-groups for outcome variables with 10 or more significant (*p* < 0.05) independent variables. Due to high variance (10^0^ to *1*0^6^ GE/ μl) of the outcome variable ‘Median GE/μl per herd’ this was logarithmically integrated (base 10) into the multivariable model.

## Results

The study includes data of 144 herds and 6450 pigs from six European countries. 90.3% of all herds sampled contained at least one positive sample. In 26.2% of sampled animals (NP, GP and FP), specific genome fragments of *L. intracellularis* were detected [3].

### Descriptive statistics

Due to the large number of variables considered during the epidemiological herd characterization, only those showing a significant correlation with the outcome variable(s) in the multivariable models are further described in detail (Table 1). Sampling was conducted between October 2017 and November 2018 with no significant differences in the number of samples per season and country (data not shown). The geographical distribution corresponded widely to the typical distribution of pig herds within the countries (data not shown). As a component concerning the environment, slatted floor in nursery was significantly less often reported in DK than in all other countries (*p* < 0.001). Furthermore, 60.0% out of the 17.5% of herds offering straw in nursery were located in DK, which made DK differing significantly from all other countries (*p* < 0.01). Before arrival of new piglets, nursery units were disinfected in 85.4% of all herds. With only 50.0% of herds doing so, the NL differed significantly from DE, ES, FR and UK (*p* < 0.05). The subsequent average down time in growing units was significantly longer in ES than in the UK (*p* < 0.01). As part of the internal biosecurity, the median number of NP per pen was determined, which turned out to be significantly higher in the UK than in all other countries (*p* < 0.001). Antimicrobial group medication (including zinc oxide) around weaning was regularly used in 51.4% and never in 32.6% of all herds. In the NL, the use of antimicrobial group medication around weaning was significantly less often noted than in DK, ES, FR and UK. ES, on the other hand, reported a much more frequent use compared to DE or FR (*p* < 0.001). Routine or occasional use of zinc oxide at weaning was declared by 41.0% of all herds. Consumption was most frequently reported for Danish (95.8%) and Spanish (79.2%) herds, which made them differ significantly from German (12.5%), French (4.2%) and Dutch (0%) herds. Also, British herds reported a significantly higher use (54.2% of herds) than French and Dutch herds (*p* < 0.001). Animals in France showed a significantly lower median age at weaning compared to DE and DK (*p* < 0.01). However, ES differed in weaning weight with lighter piglets than DE and DK. Furthermore, British NP were significantly heavier than Danish, Spanish and French NP (*p* < 0.001). Neither the mortality in NP nor the morbidity of GP at the last occurrence of diarrhoea differed significantly between countries.

**Table 1:** Variables, significant in the multivariable analysis and their country specific differences in descriptive statistics

Variables in questionnaire	Statistic	DE^a^	DK^a^	ES^a^	FR^a^	NL^a^	UK^a^	Total
% of slatted floor in nursery unit	Median	100.0	33.0	100.0	100.0	100.0	100.0	100.0
	Minimum	0.0	25.0	70.0	0.0	30.0	0.0	0.0
	Maximum	100.0	66.0	100.0	100.0	100.0	100.0	100.0
Straw in units of nursery pigs (1 = No, 2 = Yes)	Median	1.0	2.0	1.0	1.0	1.0	1.0	1.0
	Minimum	1.0	1.0	1.0	1.0	1.0	1.0	1.0
	Maximum	2.0	2.0	1.0	2.0	2.0	2.0	2.0
Frequency of disinfection in nursery (1 = Every time, 2 = Every 2nd time, 3 = Every 3rd time, 4 = Less common)	Median	1.0	1.0	1.0	1.0	1.5	1.0	1.0
	Minimum	1.0	1.0	1.0	1.0	1.0	1.0	1.0
	Maximum	4.0	4.0	1.0	1.0	4.0	2.0	4.0
Average down time in units of growing pigs (Days)	Median	3.0	2.4	7.0	4.0	3.0	2.0	3.0
	Minimum	0.3	0.0	0.0	0.0	0.0	0.0	0.0
	Maximum	14.0	14.5	84.0	10.0	14.0	8.5	84.0
Number of pigs per pen in nursery (n)	Median	40.0	34.5	27.0	25.0	26.0	100.0	35.0
	Minimum	14.0	16.0	14.0	15.0	6.0	38.0	6.0
	Maximum	275.0	50.0	225.0	50.0	200.0	220.0	275.0
Use of antimicrobials at/ around weaning (1 = Never, 2 = Sometimes, 3 = Always)	Median	1.0	3.0	3.0	2.0	1.0	3.0	3.0
	Minimum	1.0	2.0	2.0	1.0	1.0	1.0	1.0
	Maximum	3.0	3.0	3.0	3.0	3.0	3.0	3.0
Use of zinc oxide at/ around weaning (1 = No, 2 = Yes)	Median	1.0	2.0	2.0	1.0	1.0	2.0	1.0
	Minimum	1.0	1.0	1.0	1.0	1.0	1.0	1.0
	Maximum	2.0	2.0	2.0	2.0	1.0	2.0	2.0
Average age at weaning (Days)	Median	28.0	28.0	25.0	21.0	26.0	26.5	26.0
	Minimum	21.0	24.0	21.0	21.0	21.0	21.0	21.0
	Maximum	42.0	33.0	28.0	42.0	42.0	33.0	42.0
Average weight at weaning (kg)	Median	7.5	6.5	6.0	6.0	7.5	7.5	7.0
	Minimum	5.5	5.0	5.2	5.3	5.0	6.5	5.0
	Maximum	10.0	10.5	7.9	10.5	10.0	9.2	10.5
Total mortality in nursery pigs (%)	Median	2.0	2.6	2.5	2.4	2.0	2.0	2.1
	Minimum	0.5	1.0	0.8	1.0	1.0	1.0	0.5
	Maximum	5.0	6.1	7.0	8.1	6.0	6.0	8.1
Morbidity of growing pigs at last occurrence of diarrhea on farm (%)	Median	0.0	1.5	0.0	0.0	0.5	3.0	0.0
	Minimum	0.0	0.0	0.0	0.0	0.0	0.0	0.0
	Maximum	50.0	55.0	50.0	100.0	20.0	30.0	100.0

The table is based on data of 24 herds per country and qPCR results of 6450 faecal samples. Data was not normally distributed

^a^*DE* Germany, *DK* Denmark, *ES* Spain, *FR* France, *NL* the Netherlands, *UK* United Kingdom

### Univariable analysis

Subsequently, answers of 67 variables were grouped (Table 1). Twenty-seven out of these correlated significantly (*p* < 0.05) to at least one out of five outcome variables in the univariably analysis (see Additional file 2), which was performed as intermediate step before the multivariable model. After performance of correlation analysis, three variables had further to be excluded, because of collinearity resulting in 24 variables used for the multivariable model (see Additional file 2).

### Multivariable analysis

Twelve out of the 24 variables included in the general mixed model still demonstrated a significant influence on the outcome variable(s) (*p* < 0.05) (Table 2). Season showed a correlation to the number of positive FP, rising in spring (Number of positive FP/herd: Median 2 min. 0 max. 14) and reaching its maximum in summer (Median 4 min. 0 max. 15) with a subsequent decrease in autumn (Median 1 min. 0 max. 14) and minimum in winter (Median 1 min. 0 max. 12) (*p* < 0.05). As another environmental parameter, slatted flooring covering more than 78% of the surface in nursery units, was correlated to lower numbers of positive animals per herd (*p* < 0.05) and a lower median of genome equivalents determined per herd (*p* < 0.05). Use of straw in units of NP, as representative of the corresponding age categories, was correlated to a reduced number of positive FP (*p* < 0.05). Regarding internal biosecurity, disinfection performed every time before NP entered the pen, correlated with lower median genome equivalents/ herd than disinfection performed every second time or less common (*p* < 0.05). Also, an average down time of more than 5 days in units of GP was correlated to reduced general number of positive samples per herd (*p* < 0.05). Less than 30 NP per pen was correlated with both, less positive NP (*p* < 0.01) and generally fewer positive samples per herd (*p* < 0.05) as well as a lower median of detected genome equivalents/ herd (*p* < 0.05). The antimicrobial use (including zinc oxide) at/around weaning correlated with the median concentration of genome equivalents/ herd depending on the frequency of use: Never (median 0.7 min. 0 max. 3.6), sometimes (median 1.1: min. 0 max. 3.0) and always (median 0.6: min. 0 max. 3.7) (*p* < 0.05). Considering only zinc oxide, routine use at/around weaning was correlated to higher number of positive NP (*p* < 0.01) as well as higher median genome equivalents/ herd (*p* < 0.05) compared to farms using zinc oxide less frequently or not at all. Weaning with more than 28 days of age correlated with higher numbers of positive NP (number of positive NP/herd: Median 7 min: 0 max. 15) compared by the other two weaning ages of > 25 and ≤ 28 days (Median 0 min. 0 max. 15) and weaning at ≤25 days (Median 0 min. 0 max. 10) (*p* < 0.01). A weight of more than 7.8 kg at weaning, was correlated to a higher number of positive GP (*p* < 0.05) as well as general higher number of positive samples/ herd (*p* < 0.01). Mortality of 20% or more in NP was correlated to higher number of positive samples/ herd (*p* < 0.05). If the number of affected GP in last occurrence of diarrhoea (Morbidity) reached or exceeded 20%, the median number of genome equivalents/herd was higher compared to farms with less GP involved (*p* < 0.05).

**Table 2:** *P*-values of all variables included in the final multivariable analysis, analysed by generalized linear mixed model

Sub- Group	Independent variables:	Outcome variables:				
		Positive samples/ herd (n)	Median GE/μl	Positive NP / herd (n)	Positive GP/herd (n)	Positive FP/herd (n)
**Environment**	Season, in which sampling was performed					***p*** **= 0.033**
	Replacement rate/year	*p* = 0.300		*p* = 0.387		
	Flooring in units of NP	***p*** **= 0.019**	***p*** **= 0.021**	x	*p* = 0.062	
	Flooring in units of GP	*p* = 0.663	x	x		
	Straw in units of NP			x	*p* = 0.772	***p*** **= 0.048**
	Texture of feed for NP	*p* = 0.183				
**Internal biosecurity**	Occupancy in units for NP			*p* = 0.289		
	Occupancy in units for GP			x		
	Disinfection in units of NP		***p*** **= 0.017**			
	Average down time in units of GP	***p*** **= 0.025**				*p* = 0.237
	Average down time in units of FP			*p* = 0.157		
	Age group vaccinated against *Lawsonia intracellularis*					x
	Handling of runts			*p* = 0.684		
	Median number of pigs per pen in nursery	***p*** **= 0.010**	***p*** **= 0.018**	***p*** **= 0.009**		
	Use of antimicrobials at/ around weaning		***p*** **= 0.018**			
	Use of zinc oxide at/around weaning		***p*** **= 0.027**	***p*** **= 0.004**		
**Animal related**	Average age at weaning			***p*** **= 0.003**		
	Average weight at weaning	***p*** **= 0.006**			***p*** **= 0.020**	
	Average daily growth in FP	*p* = 0.209	x			
	Total mortality in NP	***p*** **= 0.021**		*p* = 0.187		
	Days between last diarrhea on farm and sampling		*p* = 0.218			
	Morbidity of NP	*p* = 0.313	*p* = 0.225	*p* = 0.321		
	Morbidity of GP		***p*** **= 0.039**			*p* = 0.407
	Morbidity of FP		*p* = 0.286			
**Number of significant variables (*p*** **< 0.05)**		**5 (10)**	**6 (11)**^**a**^	**3 (13)**^**a**^	**1 (3)**	**2 (5)**

*NP* Nursery pigs, *GP* Growing pigs, *FP* Finishing pigs, *GE* Genome equivalents, measured by quantitative Polymerase chain reaction out of faecal samples. Empty fields indicate no calculation in the multivariable model, due to a p-value of ≥0.05 in the univariable model. ‘x’ indicates an abort of the model after including this or a previous variable

^a^The deviation compared to the number of significant variables in Additional file 2, is due to the exclusion of variables with a correlation of more than 60% in the present model. Bold letters indicate a *p*-value < 0.05. The length of lines between the sub-groups is limited to the outcome variable for which they were used. Explanations on answer characteristics of the independent variables (factor levels) can be found in Additional file 1

## Discussion

With results of 6450 faecal samples, the study is based on a very large data set. However, since only 24 herds per country were examined, and these were included based on only a few inclusion and exclusion criteria [3], the herds and their statements in the questionnaires should not be assumed to be representative for the entire country.

With a total of 12 variables, whereof four correlated significantly with two or more outcome variables, a relatively large number of variables remained significant compared to other studies dealing with risk factors related to *L. intracellularis.* A possible cause for this could be the diagnostic type of test used in the present study. Quantitative PCR assays for *L. intracellularis* have only been published since 2008/9 [20, 21] and the majority of risk factor studies are therefore based on qualitative detection methods [9–11, 15, 19]. However, 6 of the 12 variables in the multivariable model correlated with the concentration of detected genome equivalents in faeces, a parameter that has so far only rarely been included in risk factor analysis. *L. intracellularis* excretion correlates with the severity of lesions in histopathology and immunohistochemistry [7, 8], which in turn are associated with its clinical relevance. Variables correlating as risk factor for such a value could indicate which factors are important in order to reduce the severity or the likeliness of an outbreak, even though the pathogen is already in the herd. This would be of great advantage for a pathogen which can be found in most pig herds in Europe [3, 22]. Another conspicuous feature is the distribution of the variables. Although many general questions on herd structure and all age categories on farm were included in the questionnaire (see Additional file 3), 9 out of 12 significantly correlating variables showed a relation to weaning or the environment in which the weaned piglets were moved. This is consistent with results of other studies [11, 14] and shows, that the spread of the pathogen can, and probably must be controlled or embanked already at this early stage. Particularly conspicuous was the positive effect of less than 30 NP per pen correlating with reduced numbers of general and nursery pigs being positive as well as less genome equivalents detected. According to the authors’ knowledge, this variable was only queried in one other study [23] with only few positive NP and indirect detection method used, which might be the reason for no significant correlation found. However, the number of animals per pen could indirectly and unequally affect the type of occupancy on farm (all in all out per pen/ compartment/ building, continuous), which could be a possible explanation for the unequal relevance in type of occupancy [11–14]. Since the studies in question make no statement about the group size in herds after weaning, this remains speculation and requires further research. Although it has rarely been proven directly in risk factor studies performed in field, the positive effect of a consistent disinfection (every time) and longer average down time (> 5 days) is biologically well comprehensible [15, 16]. The correlation of many affected NP and high mortality underline the importance of the pathogen whose high mortality is described [1]. Since the pathogen reproduces exclusively within the animal [1], a correlation between high numbers of GP with diarrhea (morbidity) and higher concentration of GE in faeces seem comprehensible. Especially since the pathogen in the current dataset was particularly frequently detected in this age category [3]. A high percentage of slatted floor, which should result in less contact with faeces, also seems biologically plausible correlating with less positive animals and GE/herd. However, this contradicts other studies in which slatted floor was identified as risk factor for an PPE outbreak [14]. Nevertheless, the presence of straw, which is often associated with the presence of concrete floor, had a positive correlation to reduced number of positive FP/herd which is in line to other studies [13, 14]. Higher weaning weight or weaning age correlated with more positive animals and GP or NP respectively. This is in contrast to another study [14] which, however did not include laboratory investigation. Perhaps, the longer time spent with the mother, a possible host of *L. intracellularis* [11, 19, 24], enables the pathogen to be transmitted to the piglets, which have fewer maternal antibodies towards the end of the suckling period [11, 25, 26]. However, this would only explain the results on NP and correlation on GP remains unsolved. As described in other studies [27–29], excretion of genome equivalents or number of animals affected did not differ between orally vaccinating and non- vaccinating herds. The vaccine was used in 14.6% (*n* = 21) of the herds at a median age of 42 days (min. 22, max. 77) at vaccination. It is noticeable, that the average age at vaccination in three studies describing a positive effect of the vaccine [30–32] was 24.7 days (min. 21, max. 32), while vaccination in studies without such findings was performed at an average age of 45.3 days (min. 33 max. 56) [27–29]. This supports the theory, that the pathogen must be prevented from spreading at the early time of weaning. Unlike other studies [17, 18], routine use of zinc oxide at/ around weaning was correlating to more positive NP and higher shedding rates of the pathogen and could therefore neither protect against infection nor multiplication of the pathogen. Of course, cause and effect cannot be completely clarified here, since herds with routine diarrhoea may also use more zinc oxide. Risk factors that favour the increased occurrence of diarrhoea could therefore be the actual cause, which is only reflected in the use of zinc oxide. This could also be the reason of the positive effects of absence of post-weaning medication [14] as shown in our study as well. Consequently, weaning pigs without the use of zinc oxide with a constant or even lower prevalence of *L. intracellularis* seems possible. This is of particular importance as the oral administration of zinc oxide to piglets will be banned in the EU in 2022 [33]. Why the number of positive animals and exclusively those of FP, increases in summer, is not described in literature nor can it be biologically explained by the authors.

## Conclusion

The evidence of only few negative herds suggest, that it is extremely difficult to exclude or eradicate *L. intracellularis* from farms. It is therefore even more important to prevent the spread and reproduction of the pathogen within the herd, which, according to this data, could be influenced by parameters that have been rarely included in risk factor analysis studies so far. Weaning and subsequent accommodation of NP seems to be of particular importance in disease prevention. Whereby especially a low number of NP per pen, the absence of zinc oxide, slatted floor of more than 78% in nursery and a weaning weight of maximal 7.8 kg seem to have a positive influence.

## Supplementary Information

**Additional file 1:.** Independent variables and their characteristics (factor levels) included in the univariable analysis after grouping answers.

**Additional file 2: ***P*- values of variables significant to at least one outcome variable in the univariable analysis.

**Additional file 3:.** Questionnaire and ANNEX which was handed over to the veterinarians who took the samples and interviewed the farmers.

## Data Availability

The datasets used and analysed during the current study are available from the corresponding author on reasonable request. The dataset supporting the conclusions of this article are included within the article and its additional files.
