# Supplementary material for: Correlation of Lawsonia intracellularis positivity in quantitative PCR and herd factors in European pig herds
Source: Porcine Health Manag. 2021 Jan 22;7:13. doi: 10.1186/s40813-021-00192-4 (PMC7821494; doi:10.1186/s40813-021-00192-4)
Supplement: Supplementary file 1 — Additional file 1:. Independent variables and their characteristics (factor levels) included in the univariable analysis after grouping answers. [file 40813_2021_192_MOESM1_ESM.docx]

**Table 1: Independent variables and their characteristics (factor levels) included in the univariable analysis after grouping answers**

| **Independent Variable (Risk factor)** | **Answer characteristics** |
| --- | --- |
| Season, in which sampling was performed* | spring/ summer/ autumn/ winter |
| Production Type | Farrow to finish farm/ 1:1 relationship |
| Number of Sows* | ≤500/ >500 |
| Replacement rate/year* | ≤ 37 %/> 37 % |
| Number of weaners* | ≤1000/ >1000 |
| Number of fattening pigs* | ≤3000/ >3000 |
| Operating location | One-Site/ Two- site/ Three- site/ Multi- site production |
| Separation of breeding and fattening herds | Yes/ No |
| Genetic* | Duroc and/ or Pietrain included/ other |
| Flooring in units of NP* | ≤ 78 % / > 78 % slatted floor |
| Flooring in units of GP¹ᵃ* | ≤ 70 % / > 70 % slatted floor |
| Flooring in units of FP¹* | ≤ 70 % / > 70 % slatted floor |
| Straw in units of NP²ᵃ | Yes/ No |
| Straw in units of GP² | Yes/ No |
| Straw in units of FP² | Yes/ No |
| Frequency of feeding in NP | Ad libitum/ 1x / 2x /3x / >3x daily |
| Frequency of feeding in GP | Ad libitum/ 1x / 2x /3x / >3x daily |
| Frequency of feeding in FP | Ad libitum/ 1x / 2x /3x / >3x daily |
| Texture of feed for NP | Liquid regular/ liquid acidified/ liquid fermented/dry regular/ dry flour texture/dry pelleted |
| Texture of feed for GP | Liquid regular/ liquid acidified/ liquid fermented/dry regular/ dry flour texture/dry pelleted |
| Texture of feed for FP | Liquid regular/ liquid acidified/ liquid fermented/dry regular/ dry flour texture/dry pelleted |
| Presentation of feeding to NP | Fully automatic/ Semi- automatic/ By hand |
| Presentation of feeding to GP | Fully automatic/ Semi- automatic/ By hand |
| Presentation of feeding to FP | Fully automatic/ Semi- automatic/ By hand |
| Hygiene on farm includes* | Change of boots (1)/ disinfectant foot bath (2)/(1) and (2) together (3)/ shower (4)/ (4) and (1) together (5)/ (4) and (2) together/ all performed |
| Occupancy in units of NP | Continuous/AiAo per pen/AiAo per room/AiAo per building |
| Occupancy in units of GP | Continuous/AiAo per pen/AiAo per room/AiAo per building |
| Occupancy in units of FP | Continuous/AiAo per pen/AiAo per room/AiAo per building |
| Cleaning in units of NP* | Every time: ground, walls, ceilings and barn alleys/ other |
| Cleaning in units of GP* | Every time: ground, walls, ceilings and barn alleys/ other |
| Cleaning in units of FP* | Every time: ground, walls, ceilings and barn alleys/ other |
| Disinfection in units of NP | Every time/ every 2^nd^ time/ every 3^rd^ time/less common |
| Disinfection in units of GP | Every time/ every 2^nd^ time/ every 3^rd^ time/less common |
| Disinfection in units of FP | Every time/ every 2^nd^ time/ every 3^rd^ time/less common |
| Average down time in units of NP* | ≤5 days/ >5 days |
| Average down time in units of GP* | ≤5 days/ >5 days |
| Average down time in units of FP* | ≤5 days/ >5 days |
| Manure management* | ≤ 3 / > 3 times per year |
| Vaccination against *Lawsonia intracellularis** | Yes/ No |
| Age group vaccinated against *Lawsonia intracellularis* * | Suckling / nursery, growing or finishing pigs |
| Deworming of NP* | Yes/ No |
| Deworming of GP* | Yes/ No |
| Deworming of FP* | Yes/ No |
| Sorting at weaning | Litter wise/ by weight/ by gender/ randomly |
| Handling of runts* | Euthanized/ placed with younger animals/ other |
| Median number of pigs per pen size* | < 30 / ≥ 30 animals |
| Use of antimicrobial at/around weaning* | never/ sometimes /always |
| Use of zinc oxide at/around weaning* | Yes/ No |
| Average age at weaning* | ≤ 25 / > 25 and ≤ 28 / > 28 days |
| Average weight at weaning* | ≤ 7.8kg/ > 7.8kg |
| Average daily growth in NP* | <400/ ≥400 and <500/ ≥500 gram per day |
| Average daily growth in GP* | ≤735/ >735 and <826/ ≥826 grams per day |
| Average daily growth in FP* | < 800 g/ ≥ 800 g and < 900 g/ ≥ 900g |
| Current problems in NP* | enteric disease/ enteric disease + other/ no enteric disease |
| Total mortality in NP* | < 4 %/ ≥ 4 % |
| Current problems in GP* | enteric disease/ enteric disease + other/ no enteric disease |
| Total mortality in GP* | <1.6 % / ≥1.6 and ≤3 %/ >3% |
| Current problems in FP* | enteric disease/ enteric disease + other/ no enteric disease |
| Total mortality in FP* | <1.6 % / ≥1.6 and ≤3 %/ >3% |
| Questions regarding last occurrence of diarrhea on farm: | |
| Days between last diarrhea on farm and sampling* | same / ≤ 10/ >10 and ≤30/ >30 day(s) |
| Morbidity of NP * | < 20 %/ ≥ 20 % |
| Morbidity of GP * | < 20 %/ ≥ 20 % |
| Morbidity of FP * | < 20 %/ ≥ 20 % |
| Lethality of NP | Number in % |
| Lethality of GP | Number in % |
| Lethality of FP | Number in % |
| Suspected diagnose * | *Lawsonia intracellularis*/ *Lawsonia intracellularis* and, or other/ other |

A star (*) indicates grouping of the variable after descriptive statistics. Variables with the same superscript numbers, correlated to more than 60 % in Spearman Correlations. The ones with a superscript ᵃ behind, were selected for further analysis. NP= Nursery pigs, GP= Growing pigs, FP= Finishing pigs
