# Supplementary material for: Correlation of Lawsonia intracellularis positivity in quantitative PCR and herd factors in European pig herds
Source: Porcine Health Manag. 2021 Jan 22;7:13. doi: 10.1186/s40813-021-00192-4 (PMC7821494; doi:10.1186/s40813-021-00192-4)
Supplement: Supplementary file 2 — Additional file 2: P- values of variables significant to at least one outcome variable in the univariable analysis. [file 40813_2021_192_MOESM2_ESM.docx]

**Table 2: P- values of variables significant to at least one outcome variable in the univariable analysis**

| **Independent variables:** | **Outcome variables:** | |  |  |  |
| --- | --- | --- | --- | --- | --- |
|  | **Positive samples (n)/ herd** | **Median GE/μl** | **Positive NP (n)/ herd** | **Positive GP (n)/ herd** | **Positive FP (n) /herd** |
| Season, in which sampling was performed | p=0.105 | p=0.187 | p=0.412 | p=0.092 | **p=0.024** |
| Replacement rate/year | **p=0.007** | p=0.153 | **p<0.001** | p=0.445 | p=0.808 |
| Flooring in units of NP | **p<0.001** | **p=0.002** | **p=0.001** | **p=0.012** | p=0.783 |
| Flooring in units of GP¹ᵃ | **p=0.045** | **p=0.008** | **p=0.033** | p=0.073 | p=0.674 |
| Flooring in units of FP¹ | p=0.070 | **p=0.007** | **p=0.021** | p=0.089 | p=0.916 |
| Straw in units of NP²ᵃ | p=0.074 | p=0.090 | **p=0.002** | **p=0.032** | **p=0.007** |
| Straw in units of GP² | p=0.606 | p=0.199 | **p=0.009** | p=0.631 | **p=0.005** |
| Straw in units of FP² | p=0.618 | p=0.072 | **p=0.015** | p=0.817 | **p=0.003** |
| Texture of feed for NP | **p=0.026** | p=0.212 | p=0.341 | p=0.389 | p=0.750 |
| Occupancy in units of NP | p=0.187 | p=0.362 | **p=0.023** | p=0.777 | p=0.239 |
| Occupancy in units of GP | p=0.292 | p=0.906 | **p=0.047** | p=0.517 | p=0.564 |
| Disinfection in units of NP | p=0.732 | **p=0.025** | p=0.787 | p=0.632 | p=0.339 |
| Average down time in units of GP | **p=0.005** | p=0.553 | p=0.063 | p=0.073 | **p=0.011** |
| Average down time in units of FP | p=0.116 | p=0.713 | **p=0.029** | p=0.620 | p=0.198 |
| Age group vaccinated against *Lawsonia intracellularis* | p=0.229 | p=0.934 | p=0.776 | p=0.770 | **p=0.049** |
| Handling of runts | p=0.179 | p=0.063 | **p=0.022** | p=0.543 | p=0.598 |
| Median number of pigs per pen in nursery | **p=0.002** | **p=0.003** | **p=0.036** | p=0.063 | p=0.108 |
| Use of antimicrobials at/around weaning | p=0.183 | **p=0.016** | p=0.077 | p=0.618 | p=0.632 |
| Use of zinc oxide at/around weaning | p=0.267 | **p=0.035** | **p=0.001** | p=0.893 | p=0.073 |
| Average age at weaning | p=0.221 | p=0.448 | **p=0.034** | p=0.399 | p=0.129 |
| Average weight at weaning | **p=0.037** | p=0.159 | p=0.713 | **p=0.007** | p=0.244 |
| Average daily growth in FP | **p=0.030** | **p=0.022** | p=0.116 | p=0.200 | p=0.722 |
| Total mortality in NP | **p=0.030** | p=0.750 | **p=0.005** | p=0.053 | p=0.961 |
| Days between last diarrhea on farm and sampling | p=0.196 | **p=0.006** | p=0.226 | p=0.174 | p=0.285 |
| Morbidity of NP | **p=0.040** | **p=0.008** | **p=0.018** | p=0.157 | **p=0.048** |
| Morbidity of GP | p=0.182 | **p=0.007** | p=0.136 | p=0.521 | p=0.372 |
| Morbidity of FP | p=0.256 | **p=0.003** | p=0.691 | p=0.269 | p=0.092 |
| **Number of significant variables (p<0.05)** | **10 (27)** | **12 (27)** | **16 (27)** | **3 (27)** | **7(27)** |

NP= Nursery pigs, GP= Growing pigs, FP= Finishing pigs, GE= Genome equivalents, measured by quantitative Polymerase chain reaction out of faecal samples. Variables with the same superscript numbers (¹ or ²), correlated to more than 60 % in the correlation analysis. The ones with a superscript ᵃ behind, were selected to stay in the model. Bold letters indicates a p-value < 0.05. Explanations on answer characteristics (factor levels) of the independent variables can be found in Supplementary material Table 1. Wilcoxon Sum Ranks test (two factor levels) or a Kruskal-Wallis ANOVA -including Dunn`s -test (three or more factor level), were performed as univariable analysis.
