# Supplementary material for: Correlation of Lawsonia intracellularis positivity in quantitative PCR and herd factors in European pig herds
Source: Porcine Health Manag. 2021 Jan 22;7:13. doi: 10.1186/s40813-021-00192-4 (PMC7821494; doi:10.1186/s40813-021-00192-4)
Supplement: Supplementary file 3 — Additional file 3:. Questionnaire and ANNEX which was handed over to the veterinarians who took the samples and interviewed the farmers. [file 40813_2021_192_MOESM3_ESM.docx]

**Questionnaire and ANNEX** which was handed over to the veterinarians who took the samples and interviewed the farmers.

Data collection form: Country/Farm-No

**Questionnaire**

*Prevalence of Lawsonia intracellularis infections in pig herds in different EU countries*

Date *(dd/mmm/yyyy)* _

**Owner:**

Name/Surname Company

Address Farm name

Country Region

Merck representative/ Vet. & Mobile phone

**Production type:**

- Farrow to finish farm
- Herd with 1:1 -or one to one to one relationship *

**1 fattening herd that receives all growing pigs from only* ***one*** *piglet producing farm* ***or*** *nursery farms that receive all piglets from* ***one*** *piglet producing farm*

**Herd size:**

Number of: Breeding sows *(n)*: replacement rate (%): /year Boars *(n)*:

Weaners *(n)*: Fattening pigs *(n)*:

**Operating location:**

- One-site production
- Two-site production → breeding and fattening herds separated:  No  Yes
- Three-site production → breeding and fattening herds separated:  No  Yes
- Multi-site production → breeding and fattening herds separated:  No  Yes

**Farm:**

|  | **Outdoor area** | |
| --- | --- | --- |
|  | No | Yes |
| Conventional |  |  |
| Label |  |  |
| Organic |  |  |

**If there is an outdoor area:**

***(multiple answers possible)***

- - Concrete floor
  - Soil
    - Regular pasture drive
    - Always the same pasture

**Genetic:** *(e.g. PIC, Dan Breed,…)*

**Flooring:**

|  | Slatted floor  quota/amount in % | | Concrete floor  in % | | Straw | |
| --- | --- | --- | --- | --- | --- | --- |
|  |  |  |  |  | No | Yes |
| **Nursery pigs** (ca. 10- 25kg) |  | |  | |  |  |
| **Growing pigs** (ca. 25- 40kg) |  |  |  |  |  |  |
| **Finishing pigs** (ca. 40- 100kg) |  |  |  |  |  |  |

Page **1** of **7**

**Feeding:**

|  | **Nursery pigs** (ca. 10- 25kg) | **Growing pigs** (ca. 25- 40kg) | **Finishing pigs** (ca. 40- 100kg) |
| --- | --- | --- | --- |
| Restrictive: | 1x 2x 3x >3x daily   -    | 1x 2x 3x >3x daily   -    | 1x 2x 3x >3x daily   -    |
| Ad libitum |  |  |  |

|  | **Nursery pigs** (ca. 10- 25kg) | | **Growing pigs** (ca. 25- 40kg) | **Finishing pigs** (ca. 40- 100kg) | |
| --- | --- | --- | --- | --- | --- |
| Liquid feeding | | | | | |
| - Regular - Acidified - Fermented | |  |  |  |  |
|  |  |  |  |  |  |
|  |  |  |  |  |  |
| Dry feed | | | | | |
| - Regular - Flour textured - Pelleted | |  |  |  |  |
|  |  |  |  |  |  |
|  |  |  |  |  |  |

|  | **Nursery pigs** (ca. 10- 25kg) | **Growing pigs** (ca. 25- 40kg) | **Finishing pigs** (ca. 40- 100kg) |
| --- | --- | --- | --- |
| Full- automatic |  |  |  |
| Semi- automatic |  |  |  |
| By hand |  |  |  |

**Hygiene:**

Hygiene lock at the farm entry:  No  Yes

**If yes**: hygiene lock consisting of: ***(multiple answers possible)***

- herd specific clothing of the company`s staff  coat/ clothes  boots/ shoes
- for visitors: herd specific  coat/ clothes  boots/ shoes
- change of boots in the barns  every barn / stable
  - every compartment
- disinfectant containing foot baths  every barn / stable
  - every compartment
- shower before entry 

**Equipment:**

Use of the same equipment* in several rooms  No  Yes  sometimes

**for example: paddle, mobile gates, dustpan*

Use of thoroughly cleaned equipment in cleaned and disinfected pens/stables  No  Yes

**Rodent control:**

- Carried out if necessary
- Always carried out prophylactically
- Not performed

**Occupancy:**

|  | **Continually** | **All in- all out** | | |
| --- | --- | --- | --- | --- |
|  |  | Per pen | Per room/ compartment | Per building |
| Nursery pigs (ca. 10- 25kg) |  |  |  |  |
| Growing pigs (ca. 25- 40 kg) |  |  |  |  |
| Finishing pigs (ca. 40- 100kg) |  |  |  |  |

**Cleaning:** *(multiple answers possible)*

|  | Every time | Every 2^nd^ time | Every 3^rd^ time | Less common |
| --- | --- | --- | --- | --- |
| **Nursery pigs** (ca. 10- 25kg) | | | | |
| room- clean/ well- swept |  |  |  |  |
| high- pressure cleaner  warm  cold water   - Ground - Walls - Ceiling - Barn alleys |  |  |  |  |
|  |  |  |  |  |
|  |  |  |  |  |
|  |  |  |  |  |
| **Growing pigs** (ca. 25- 40kg) | | | | |
| room- clean/ well- swept |  |  |  |  |
| high- pressure cleaner  warm  cold water   - Ground - Walls - Ceiling - Barn alleys |  |  |  |  |
|  |  |  |  |  |
|  |  |  |  |  |
|  |  |  |  |  |
| **Finishing pigs** (ca. 40- 100kg) | | | | |
| room- clean/ well- swept |  |  |  |  |
| high- pressure cleaner  warm  cold water   - Ground - Walls - Ceiling - Barn alleys |  |  |  |  |
|  |  |  |  |  |
|  |  |  |  |  |
|  |  |  |  |  |

**Disinfection:**

|  | Every time | Every 2^nd^ time | Every 3^rd^ time | Less common |
| --- | --- | --- | --- | --- |
| **Nursery pigs** (ca. 10- 25kg) |  |  |  |  |
| **Growing pigs** (ca. 25- 40kg) |  |  |  |  |
| **Finishing pigs** (ca. 40- 100kg) |  |  |  |  |

Most commonly used disinfecting agent? ***(multiple answers possible)***

- - Quaternary ammonium compounds
  - Pyrovidone- iodide
  - Potassium- peroxymonosulfate
  - Phenoderivate
  - Alcohol
  - Organic acids
  - Guanidine
  - Other:

Average down time (*time between disinfection and arrival of new pigs*)?

- **Nursery pigs** (ca. 10- 25kg) days
- **Growing pigs** (ca. 25- 40kg) days
- **Finishing pigs** (ca. 40- 100kg) days

**Manure management**

How frequently do you empty the manure under the floor? /per year

[**Immunoprophylaxis**](http://www.dict.cc/englisch-deutsch/immunoprophylaxis.html)**:**

Suckling pigs **Use codes on the enclosed sheet (ANNEX)*

| Vaccine  (*Code)* | Age at vaccination/ frequency | Application  *(i.m.; s.c.; p.o.; i.d.)* |
| --- | --- | --- |
| *e.g.: F* | *14 days of age* | *p.o. (drenching)* |

*i.m. = intramuscular s.c. = subcutan i.d. = intradermal p.o. = per os (water)/ (feed)/ (drenching)*

Nursery, growing and finishing pigs **Use codes on the enclosed sheet (ANNEX)*

| Vaccine  (Code*)* | Time frame | Application  *(i.m.; s.c.; p.o.; i.d.)* |
| --- | --- | --- |
|  |  |  |

*i.m. = intramuscular s.c. = subcutan i.d. = intradermal p.o. = per os (water)/ (feed)/ (drenching)*

**Health status:**

| **Sows**: Deworming:   - No  Yes   - time-oriented  1x/ year     - 2x/ year   - production-oriented | | **Piglets**: Deworming:   - No  Yes   days of nursery period: Treatment against coccidian parasites:   - No  Yes | |
| --- | --- | --- | --- |
| **Nursery pigs** (ca. 10- 25kg)  Deworming:   - No - Yes → days of fattening   period: | **Growing pigs** (ca. 25- 40kg) | | **Finishing pigs** (ca. 40- 100kg) |
|  | Deworming:   - No - Yes → days of fattening   period: | | Deworming:   - No - Yes → days of fattening   period: |

**Nursery pigs:**

Age at weaning days

Average weight at weaning kilogram

Strategy of sorting in the nursery: *(multiple answers possible)*

- - Litter wise
  - Sorted by weight
  - By gender
  - Randomly

Median number of pigs per pen size:

**Routine treatment during / after weaning *:** **between first and last day of nursery period*

**Antimicrobials**:

- 1 out of 4 batches
- 2 out of 4 batches
- 3 out of 4 batches
- More frequently than 3 out of 4 batches
- Always
- Never

**Medication route:**

| - Feed - Water - Injection | - Injection and feed - Injection and water - Feed and water |
| --- | --- |

Antimicrobial/ Active ingredient: *(*Code)*:

**Use the Code on the enclosed sheet (ANNEX)*

Dosage mg/kg body weight Duration of treatment days

|  | **Average daily growth** *(gram)* | |
| --- | --- | --- |
| **Nursery pigs** (ca. 10- 25kg) |  | |
| **Growing pigs** (ca. 25- 40kg) |  |  |
| **Finishing pigs** (ca. 40- 100kg) |  |  |

**Runts: *(multiple answers possible)***

| - Stay in the group until slaughter - Slaughtered earlier - Separated | - Euthanized - Placed with younger animals - There are no runts |
| --- | --- |

**Current problems in the herd: *(multiple answers possible)***

| **Clinical signs** | **Nursery pigs**  (ca. 10- 25kg) | **Growing pigs**  (ca. 25- 40kg) | **Finishing pigs**  (ca. 40- 100kg) |
| --- | --- | --- | --- |
| **Enteric diseases** |  |  |  |
| **Respiratory** |  |  |  |
| **Central nervous system** |  |  |  |
| **Locomotor system** |  |  |  |
| **Other clinical signs?** |  |  |  |
| **Total mortality (%)** |  |  |  |

**The following questions all regard to the last occurrence of diarrhea on the farm:**

Date of last occurrence of diarrhea in the herd? (dd/mmm/yyyy)

Which age groups were affected?

- **Nursery pigs** (ca. 10- 25kg)
- **Growing pigs** (ca. 25- 40kg)
- **Finishing pigs** (ca. 40- 100kg)

Distribution of the affected animals  only 1 batch affected  several batches affected

- - batches continually affected  coincidentally What percentage of animals became diseased (morbidity)?
- **Nursery pigs** (ca. 10- 25kg) %
- **Growing pigs** (ca. 25- 40kg) %
- **Finishing pigs** (ca. 40- 100kg) %

What percentage of diseased animals died (lethality)?

- **Nursery pigs** (ca. 10- 25kg) %
- **Growing pigs** (ca. 25- 40kg) %
- **Finishing pigs** (ca. 40- 100kg) %

How did faeces mostly look during the last outbreak of diarrhea?*

**Use scores on the enclosed sheet (ANNEX)*

|  | **Score ranges from … to ….** | **Most of the pigs showed a score of …** |
| --- | --- | --- |
| **Nursery pigs** (ca. 10- 25kg) |  |  |
| **Growing pigs** (ca. 25- 40kg) |  |  |
| **Finishing pigs** (ca. 40- 100kg) |  |  |

Suspected diagnosis *(tentative)*:

Which diagnostic tests were performed? ***(multiple answers possible)***

| - ELISA - conventional PCR - real time PCR | - Histology - Culture - Flotation | - Immunofluorescence - None - Other |
| --- | --- | --- |

How many samples were taken?

**Findings in diagnostic tests: *(multiple answers possible)***

| - *Brachyspira hyodysenteriae* - *Brachyspira pilosicoli* - *Clostridium perfringens* - *Clostridium novyi* - *Coccidia* - *Coronavirus* - *Escherichia coli* | - *Lawsonia intracellularis* - Parasites - *Rotavirus* - *Salmonella spp.* - None - Other: |
| --- | --- |

Confirmation of the suspected diagnosis?  No  Yes Treatment of affected animals?  No  Yes

- - Antimicrobials
  - Other:

Which Antimicrobials /active ingredients were used? **Use codes on the enclosed sheet (ANNEX)*

| Antimicrobial/Active ingredient  *(Code)* | Beginning of the treatment  *(dd/mmm/yyyy)* | End of the treatment  *(dd/mmm/yyyy)* | Route  *(p.o.; p.i.; co.)* |
| --- | --- | --- | --- |
| *e.g.: 29* | *05/Feb/2017* | *11/Feb/2017* | *p.o.(water)* |

*p.o. = per os (feed)/ (water) p.i. = per injection co.= combination of injection and per os*

Which medications have been applied to batches of the three categories of pigs within the last 6 months and why?

|  | Indication  *(Code)* | 1^st^ day of administration  *(dd/mmm/yyyy)* | Last day of administration  *(dd/mmm/yyyy)* | Antimicrobial agents/  Active ingredient | Route  *(p.o.; p.i.; co.)* |
| --- | --- | --- | --- | --- | --- |
| Nursery pigs (ca. 10- 25kg) | *e.g.: 2* | *01/Jan/2017* | *05/Jan/2017* | *24* | *p.o. (feed)* |
| Growing pigs (ca. 25- 40kg) |  |  |  |  |  |
| Finishing pigs (ca. 40- 100kg) |  |  |  |  |  |

*Codes:*

- *Indication: 1=* enteric diseases *2= respiratory 3= central nervous system 4= locomotor system 5= other*
- *Antimicrobial agents: see enclosed sheet (ANNEX)*
- *Route: p.o. = per os- (water)/ (feed) p.i. = per injection co.= combination of injection and per os*

**ANNEX**

*Prevalence of Lawsonia intracellularis infections in pig herds in different EU countries*

**Immunoprophylaxis**

| **Vaccine** | **Code** |
| --- | --- |
| *Actinobacillus pleuropneumoniae* | A |
| *Bordetella bronchiseptica* | B |
| *Coronavirus* | C |
| *Escherichia coli* | D |
| *Haemophilus parasuis* | E |
| *Lawsonia intracellularis* | F |
| *Mycoplasma hyopneumoniae* | G |
| *Pasteurella multocida* | H |
| *Porcine circovirus type 2* | I |
| *Porcine reproductive and respiratory syndrome virus* | J |
| *Salmonella* spp*.* | K |
| *Streptococcus suis* | L |
| *Swine herpesvirus type 1* | M |
| *Swine influenza virus* | N |
| Autogenous vaccine and others* | O |

**Please write the Code (O), the trade name and against which pathogen the vaccine is targeted*

**Score for assessing the faeces during the last outbreak of diarrhea**

| **Score** | **Description** | **Example picture** |
| --- | --- | --- |
| **1** | Diarrhea with blood or mucus | 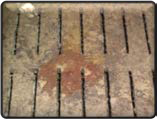 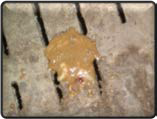 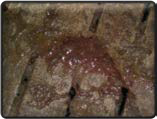 |
| **2** | Liquid (diarrhea) | 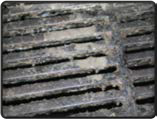 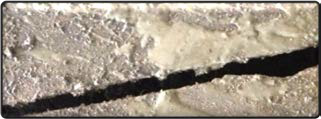 |
| **3** | Pasty | 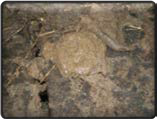 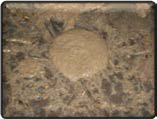 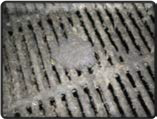 |
| **4** | Formed, soft | 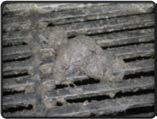 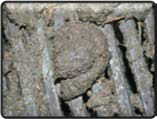 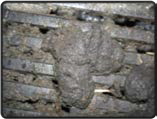 |

**Antimicrobials/ Active ingredients**

| **Antimicrobial/ Active ingredient** | **Code** |
| --- | --- |
| Amoxicillin | 1 |
| Amoxicillin/ Clavulanic acid | 2 |
| Ampicillin | 3 |
| Apramycin | 4 |
| Cefquinome | 5 |
| Ceftiofur | 6 |
| Cephalothin | 7 |
| Chlortetracycline | 8 |
| Clindamycin | 9 |
| Colistin | 10 |
| Colistin sulphate/ Polymyxin B | 11 |
| Danofloxacin | 12 |
| Enrofloxacin | 13 |
| Erythromycin | 14 |
| Florfenicol | 15 |
| Gentamycin | 16 |
| Josamycin | 17 |
| Lincomycin | 18 |
| Neomycin | 19 |
| Oxytetracycline | 20 |
| Penicillin | 21 |
| Spectinomycin | 22 |
| Sulfonamid + Trimethoprim | 23 |
| Tetracyclin | 24 |
| Tiamulin | 25 |
| Tilmicosin | 26 |
| Trimethoprim sulfamethoxazole | 27 |
| Tulathromycin | 28 |
| Tylosin | 29 |
| Tylvalosin | 30 |
| Valnemulin | 31 |
| **Zinc oxide** | 32 |
